# The role of plastic reconstructive surgery in surgical reconstruction of soft tissue defects after resection of musculoskeletal tumors

**DOI:** 10.1016/j.jpra.2026.01.046

**Published:** 2026-01-31

**Authors:** Parthena Deskoulidi, Vasileios Kontogeorgakos, Olga Savvidou, Konstantinos Benetatos, Panagiotis Gavriil, Stavros Goumenos, Panayiotis Papagelopoulos

**Affiliations:** a Department of Plastic and Reconstructive Surgery, Kat Hospital, Athens, Greece; b First Department of Orthopaedic Surgery, School of Medicine, National and Kapodistrian University of Athens, “Attikon” University General Hospital, Athens, Greece; c Department of Plastic and Reconstructive Surgery, 401 Military Hospital of Athens, Greece

**Keywords:** Musculoskeletal tumors, Orthoplastic approach, Sarcomas, Reconstruction, DIEP flap, ALT flap

## Abstract

**Background:**

Musculoskeletal tumor resections often result in large soft tissue defects that require complex reconstructive surgery to restore function and appearance. An interdisciplinary “orthoplastic” approach—coordinating orthopedic and plastic surgical expertise—aims to ensure durable wound coverage, minimize complications, and facilitate timely adjuvant therapies, ultimately improving functional recovery and oncologic outcomes.

**Methods:**

We retrospectively analyzed patients (2013-2020) at the First Department of Orthopedic Surgery, Athens University Medical School, “Attikon” University General Hospital. Focusing on sarcomas primarily in the lower extremities, pelvis, and sacrum, 56 patients underwent wide local resection and plastic reconstruction. Twenty-three of these 56 patients (41%) required wide local excision and pedicle or free flap reconstruction. Data included demographics, tumor characteristics, surgical details, and postoperative outcomes. Reconstructive technique choice depended on defect size, location, critical structure exposure, and prior radiation.

**Results:**

The orthoplastic approach, with meticulous planning and diverse reconstructive options, achieved acceptable morbidity and favorable functional and oncologic outcomes. This strategy ensured clear tumor margins with durable soft tissue coverage, protecting vital structures and minimizing wound complications, especially in limb-salvage cases requiring adjuvant radiotherapy. Our institutional data confirmed tailored reconstruction facilitated successful defect closure and timely adjuvant therapy.

**Conclusion:**

A collaborative orthoplastic approach is pivotal for soft tissue reconstruction after musculoskeletal tumor resection, improving limb preservation, outcomes, and reducing complications. While immediate reconstruction with well-vascularized tissue is beneficial, further research is needed to quantify its impact on quality of life and recurrence, and to establish evidence-based guidelines. Our experience highlights the frequent need for plastic surgery in extremity/pelvic sarcomas and the value of a flexible, tailored approach.

## Introduction

The surgical management of musculoskeletal tumors frequently results in extensive soft tissue defects, requiring complex reconstructive techniques to restore both function and aesthetics. These tumors constitute a heterogeneous group of neoplasms originating from bone and soft tissues, representing only 0.2–0.5% of all malignancies across age groups but presenting a major therapeutic challenge due to their anatomical diversity and biological behavior. They are broadly categorized as benign or malignant, with significant variations in incidence, histopathological features, and geographic distribution.^1^^,^^2^

The World Health Organization (WHO) provides a comprehensive classification system for bone and soft tissue tumors based on cellular origin.^3^ Pathologic diagnosis relies primarily on morphology, supplemented by immunohistochemistry and molecular analysis.^4^ Expert review is essential, as diagnostic discrepancies between general pathologists and sarcoma specialists remain common.^4^ Malignant musculoskeletal tumors, collectively termed *sarcomas*, account for <1% of adult and approximately 15% of pediatric malignancies.^5^ The WHO currently recognizes around 50 histologic types of soft tissue sarcomas (STS) and 30 types of bone sarcomas.^4^

Among malignant bone tumors, osteosarcoma is the most frequent, comprising approximately 21.5% of cases, followed by Ewing’s sarcoma (16%) and chondrosarcoma (4.5%).^6^ Rarer variants—such as chondroblast osteosarcoma, fibrosarcoma, and synovial sarcoma—collectively account for about 7.5%.^6^ Benign bone tumors are significantly more common, with osteochondroma representing 63.9% of cases, followed by enchondroma (10.8%), giant cell tumor (9.9%), and osteoid osteoma (6.6%).^7^ Soft tissue tumors, meanwhile, are dominated by liposarcomas and leiomyosarcomas, though benign soft tissue masses occur far more frequently than malignant ones.^1^

Epidemiological studies show that sarcoma incidence and distribution vary regionally. In Europe, the annual incidence of adult-type soft tissue and visceral sarcomas (excluding GISTs) is estimated at 4–5 per 100,000, while most histotypes occur at fewer than 2 per million.^4^ Between 2000 and 2018, the global prevalence of malignant bone tumors increased from 0.00069% to 0.00749%, reflecting improved diagnostic capabilities.^8^ Benign bone tumors, often asymptomatic, remain underreported.^3^ Studies from the Middle East reveal regional variations in tumor prevalence—for instance, osteochondroma is more common than Ewing sarcoma in some populations.^3^^,^^6^ Similar population-based research from Canada, Italy, Turkey, and Iran highlights significant geographic heterogeneity in musculoskeletal tumor epidemiology.^9^, ^10^, ^11^, ^12^

## The orthoplastic approach

Plastic and reconstructive surgery plays a pivotal role in the interdisciplinary management of musculoskeletal tumors, ensuring both oncologic safety and functional preservation.^13^ Historically, orthopedic oncologists and plastic surgeons often operated independently. However, growing recognition of the benefits of collaborative care led to the development of the *orthoplastic approach* in the early 1990s—a model integrating orthopedic and plastic surgical expertise to enhance outcomes, particularly in limb-salvage scenarios.^14^

This coordinated strategy directly impacts both functional recovery and oncologic prognosis by achieving stable wound healing, minimizing complications, and enabling early initiation of adjuvant therapy.^14^ While musculoskeletal oncology focuses on complete tumor excision with clear margins, orthoplastic collaboration ensures durable soft tissue coverage, functional repair, and satisfactory aesthetic results.^14^ The approach also provides vital protection for neurovascular structures, prosthetic implants, and bone, reducing the risk of infection and implant exposure.

Modern reconstructive options range from simple skin grafts to complex microsurgical free tissue transfers and pedicled perforator flaps, allowing tailored solutions for each clinical scenario.^14^ Immediate, one-stage reconstruction by a multidisciplinary team has been shown to be feasible and safe, reducing morbidity, optimizing limb salvage, and improving overall patient outcomes.^14^ Preoperative imaging and vascular mapping are crucial for flap planning and donor-site selection, particularly in irradiated or scarred fields.^14^

Well-vascularized flap reconstruction is particularly beneficial for patients requiring adjuvant radiotherapy, as it promotes healing and reduces wound complications.^14^^,^^15^ Despite these advantages, the optimal timing of reconstruction in relation to radiotherapy—pre- or postoperative—remains an area of active investigation.^15^ Tension-free closure using vascularized tissue is generally recommended to mitigate the deleterious effects of radiation.^14^^,^^16^ Innovations such as chimeric flaps, which combine soft tissue and bone, offer additional versatility for complex reconstructions, including head and neck cases.^17^

## Role of imaging and multidisciplinary care

Magnetic resonance imaging (MRI) remains the gold standard for evaluating primary soft tissue sarcomas of the trunk, pelvis, and extremities due to its superior soft tissue contrast and ability to define tumor relationships to neurovascular structures.^18^ MRI also plays a critical role in detecting local recurrence, with reported sensitivity and specificity as high as 92% and 98%, respectively.^19^ In ambiguous cases, FDG-PET/CT can provide additional diagnostic clarity. Biopsy, ideally guided by imaging and performed after multidisciplinary discussion, is essential for histologic confirmation.^19^

Optimal management requires a dedicated multidisciplinary team (MDT) including pathologists, radiologists, orthopedic oncologists, plastic surgeons, radiation and medical oncologists, and, when applicable, pediatric oncologists or organ-specific specialists. Collaboration within reference centers or networks enhances adherence to best-practice guidelines and improves relapse-free survival.^20^^,^^21^

Despite increasing adoption, the literature remains limited regarding the specific contribution of plastic reconstructive surgery following sarcoma excision and the consolidated outcomes of orthoplastic flap management. High-quality, multi-institutional studies are needed to establish evidence-based recommendations and quantify long-term benefits on functional outcomes, recurrence, and quality of life.

The present study aims to highlight and reinforce the value of the orthoplastic strategy in optimizing surgical care and improving the quality of life of patients with musculoskeletal tumors.

## Materials and methods

### Study design and setting

We conducted a retrospective, single-institution study at the First Department of Orthopaedic Surgery, Athens University Medical School, “Attikon” University General Hospital. The study period was 2013–2020 and focused on patients undergoing primary musculoskeletal tumor resection with immediate or delayed flap-based reconstruction within an orthoplastic care pathway. All sarcoma patients were surgically treated by the senior author (PJP).

### Ethics

This Study involving human participants was reviewed and approved by the Ethics Committee of “Attikon” University General Hospital (Approval No.EBD2Ι4/30-03-2022).

### Patient population

Eligible patients were those who (i) underwent wide local excision of a musculoskeletal tumor by the orthopedic oncology team and (ii) required plastic surgical reconstruction for resultant soft-tissue defects. Patients treated by primary closure alone were excluded. During the study window, 56 patients underwent wide local excision and plastic reconstruction, of whom 23 (41%) required pedicle or free flap reconstruction and formed the analytic cohort.

### Data sources and variables

Data were abstracted from electronic medical records and operative reports, including:

• Demographics and comorbidities.
• Tumor characteristics: histology, site, size, grade/stage, prior treatments (radiotherapy/chemotherapy).
• Surgical details: resection type/margins, timing of reconstruction (immediate vs delayed), flap type (pedicled vs free), recipient vessels, and adjuncts (dermal matrices, VAC therapy).
• Postoperative outcomes: flap failure/compromise, wound complications, reoperations, time to adjuvant therapy, and length of stay.
• Functional/aesthetic outcomes and local/distant recurrence where available.

### Preoperative evaluation and planning

All cases were discussed in a multidisciplinary tumor board including orthopedic and plastic surgeons, radiology, pathology, and medical/radiation oncology.^21^ Imaging included MRI as the primary modality for local staging and neurovascular mapping, with PET/CT selectively for problem-solving or metastatic assessment.^19^, ^20^, ^21^ Biopsy tract planning followed MDT consensus to avoid compromising definitive resection and reconstruction.^21^ Vascular mapping (Doppler/CTA) was obtained selectively to guide perforator-based and free-flap planning.^14^

### Surgical oncologic management

The oncologic strategy was wide tumor resection with margins per contemporary sarcoma principles, aiming for limb preservation when feasible. Margin goals were not compromised for soft-tissue closure; instead, reconstruction was planned to enable margin-adequate resections (“oncologic first” doctrine).^14^

### Reconstructive strategy (orthoplastic algorithm)

Reconstruction timing and technique were tailored to defect size, location, exposure of critical structures, and prior radiotherapy.^14^^,^^22^^,^^23^ Immediate reconstruction with well-vascularized tissue was preferred to support wound healing and timely adjuvant therapy.^14^^,^^22^ When final pathology was pending or additional margin control was anticipated, negative-pressure wound therapy and/or dermal matrices were used as a temporizing strategy before definitive coverage.^22^

### General principles

1. Prioritize durable, tension-free coverage with vascularized tissue in irradiated or high-risk beds.^14^, ^15^, ^16^
2. Protect exposed bone, tendons, neurovascular structures, and implants.
3. Choose pedicled options when reliable and anatomically suited; use free tissue transfer for large, composite, or poorly vascularized defects.
4. Plan incisions and recipient vessels to preserve future oncologic options.

### Small/superficial defects

Primary closure when feasible; split-thickness skin grafts used selectively, avoiding fields anticipating adjuvant radiotherapy due to higher risks of breakdown and unstable scars.^14^^,^^24^^,^^25^

### Anatomic pathways (summary)

• **Head & Neck:** Pedicled pectoralis major, latissimus dorsi, and supraclavicular artery perforator flaps; free flaps (e.g., DIEP, ALT) for large or previously irradiated fields.^24^^,^^25^
• **Upper Limb:** Local/regional options (radial forearm, reverse radial/ulnar, posterior interosseous, reverse lateral arm; FCU/brachioradialis for elbow). Free flaps (ALT, gracilis, serratus anterior, scapular/parascapular, latissimus) for extensive defects or composite needs.^22^^,^^26^
• **Thigh/Pelvis:** Pedicled options (ALT, vastus lateralis, gracilis, sartorius, gluteus maximus; VRAM/transpelvic VRAM for pelvic/sacral coverage). Free flaps as indicated for large defects or implant coverage.^23^^,^^27^
• **Leg/Foot & Ankle:** Gastrocnemius (upper third), soleus (middle third), peroneus brevis and perforator flaps for small–medium defects; free flaps for extensive exposure of bone, tendons, or vessels.^27^^,^^28^

### Perioperative radiotherapy and timing

When radiotherapy was indicated, timing (pre- vs postoperative) followed MDT decision-making. For irradiated fields, vascularized flap reconstruction was favored to mitigate wound morbidity; tension-free closure was a standard goal.^14^, ^15^, ^16^ The timing question remains an area of ongoing investigation.^15^

### Outcomes and definitions

• **Primary endpoints:** major wound complication (requiring reoperation, prolonged VAC, or readmission), total/partial flap loss, and time to initiation of adjuvant therapy.
• **Secondary endpoints:** length of stay, return to theater for non-flap complications, functional outcomes (documented limb salvage, range of motion/ambulation where available), aesthetic satisfaction (noted in clinic), and oncologic outcomes (local recurrence, metastasis) at last follow-up.

### Statistical approach

Given the cohort size, analyses were descriptive (counts, percentages, medians/IQR). Where appropriate, subgroup summaries were stratified by anatomic site, flap category (pedicled vs free), and radiation exposure. No inferential statistics were planned a priori.

## Results

### Patient demographics and tumor characteristics

Between 2013 and 2020, 23 of 56 patients (41%) undergoing wide local excision for musculoskeletal tumors at the 1st Department of Orthopaedic Surgery, “Attikon” University General Hospital, required plastic surgical reconstruction. The cohort included 12 men (52%) and 11 women (48%), with a mean age of 51.7 years (range 28–74). The majority of tumors were located in the lower extremities (*n* = 13, 56.5%), followed by the sacrum/pelvis (*n* = 5, 21.7%), trunk (*n* = 2, 8.7%), upper extremities (*n* = 2, 8.7%), and head/neck region (*n* = 1, 4.3%). Histologically, the most frequent diagnoses were liposarcoma (*n* = 8, 34.8%) and chondrosarcoma (*n* = 5, 21.7%), followed by chordoma (*n* = 5, 21.7%), malignant peripheral nerve sheath tumor (MPNST, *n* = 2, 8.7%), synovial sarcoma (*n* = 2, 8.7%), and myxofibrosarcoma (*n* = 1, 4.3%).

Table 1 summarizes tumor types, locations, and reconstructive techniques applied.

**Table 1:** Distribution of musculoskeletal tumor types and reconstructive techniques.

Tumor type	Anatomical location	Number of patients (*n*)	Reconstructive technique
Liposarcoma	Thigh/Trunk	8	Pedicled flap (6 thigh), Latissimus dorsi (2 trunk)
Chondrosarcoma	Thigh/Pelvis	5	Pedicled thigh flaps, Vastus lateralis/VRAM
Chordoma	Sacrum/Pelvis	5	3 Gluteal flaps, 2 Transpelvic VRAM flaps
MPNST	Thigh	2	Pedicled flaps
Synovial Sarcoma	Upper limb	2	Free anterolateral thigh (ALT) flaps
Myxofibrosarcoma	Head and neck	1	Free DIEP flap
Total		23	

### Reconstructive techniques

Pedicled flaps were employed in 13 patients (56.5%) and free flaps in 6 patients (26.1%). Four patients (17.4%) underwent composite or staged procedures involving dermal matrices or vacuum-assisted closure (VAC) prior to definitive coverage.

• Pedicled flaps included vastus lateralis, gluteus maximus, sartorius, and VRAM flaps, primarily for thigh and pelvic defects.
• Free flaps comprised anterolateral thigh (ALT) flaps, DIEP flaps, and latissimus dorsi flaps, utilized for large or irradiated fields, especially in head/neck, upper limb, and trunk reconstructions.

All reconstructions achieved stable wound closure and allowed initiation of adjuvant therapy within 4–6 weeks postoperatively, depending on histology and oncologic plan.

**Figure 1:**
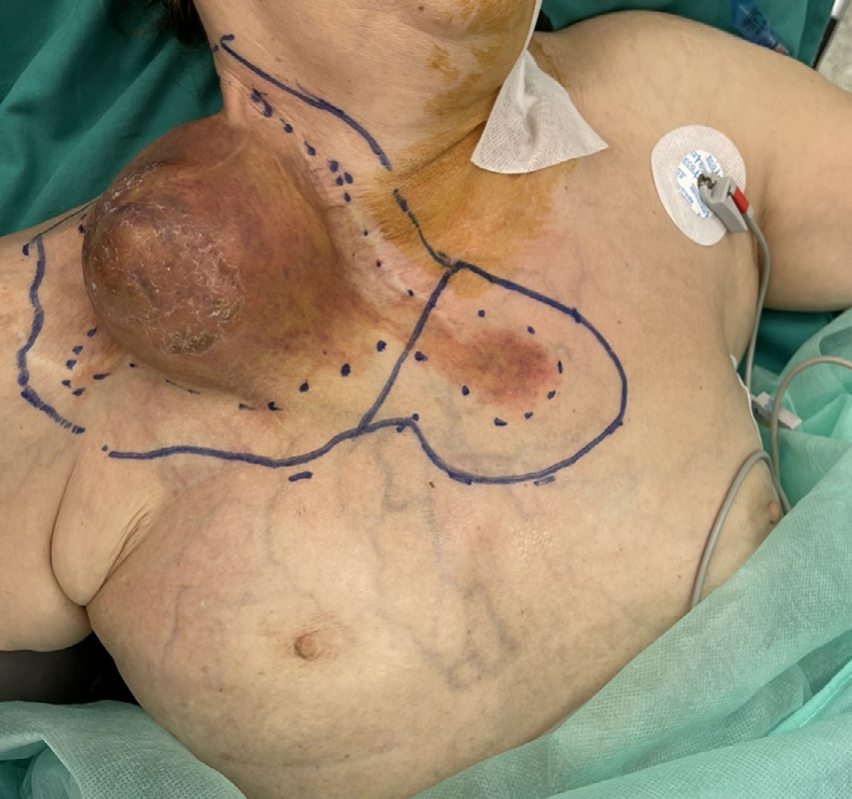
Head and neck myxofibrosarcoma wide excision incision.

**Figure 2:**
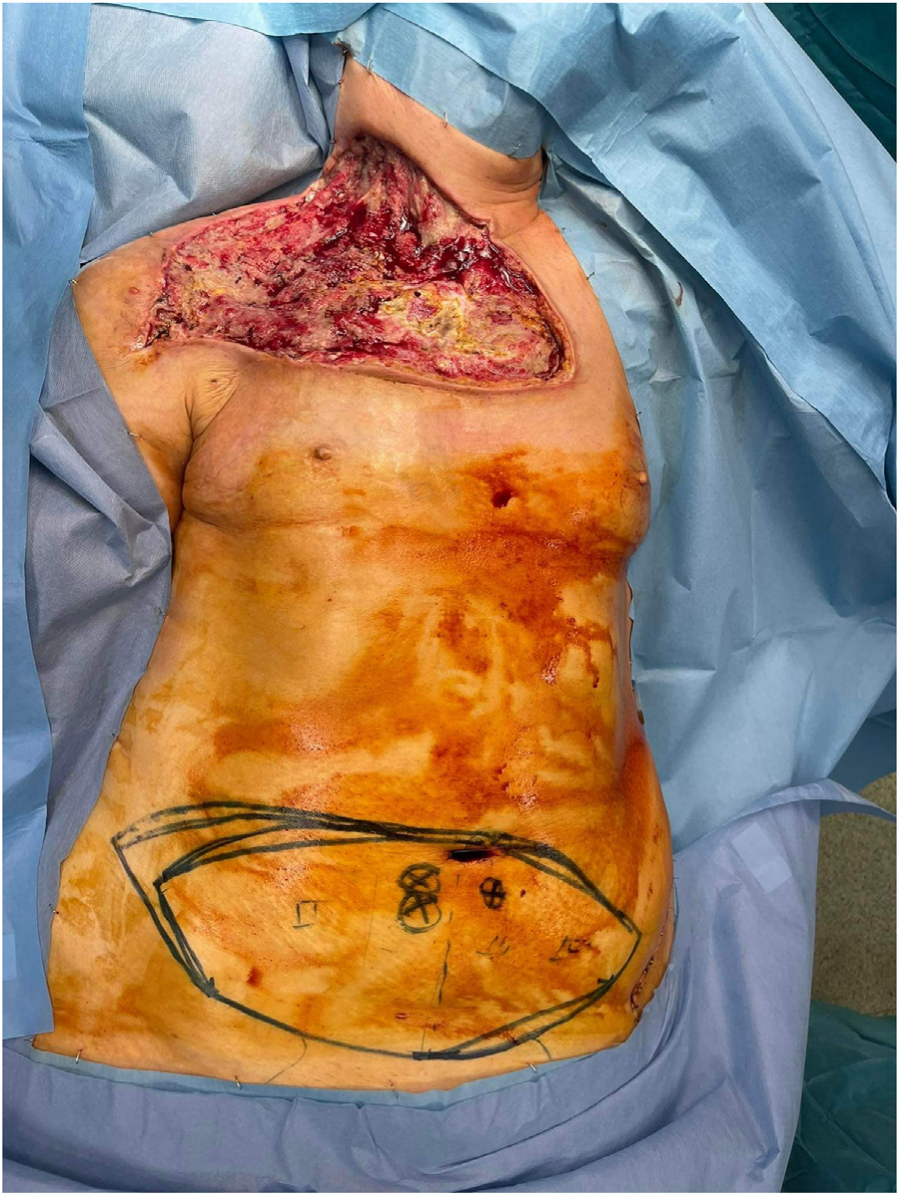
Bipedicled DIEP flap markings.

**Figure 3:**
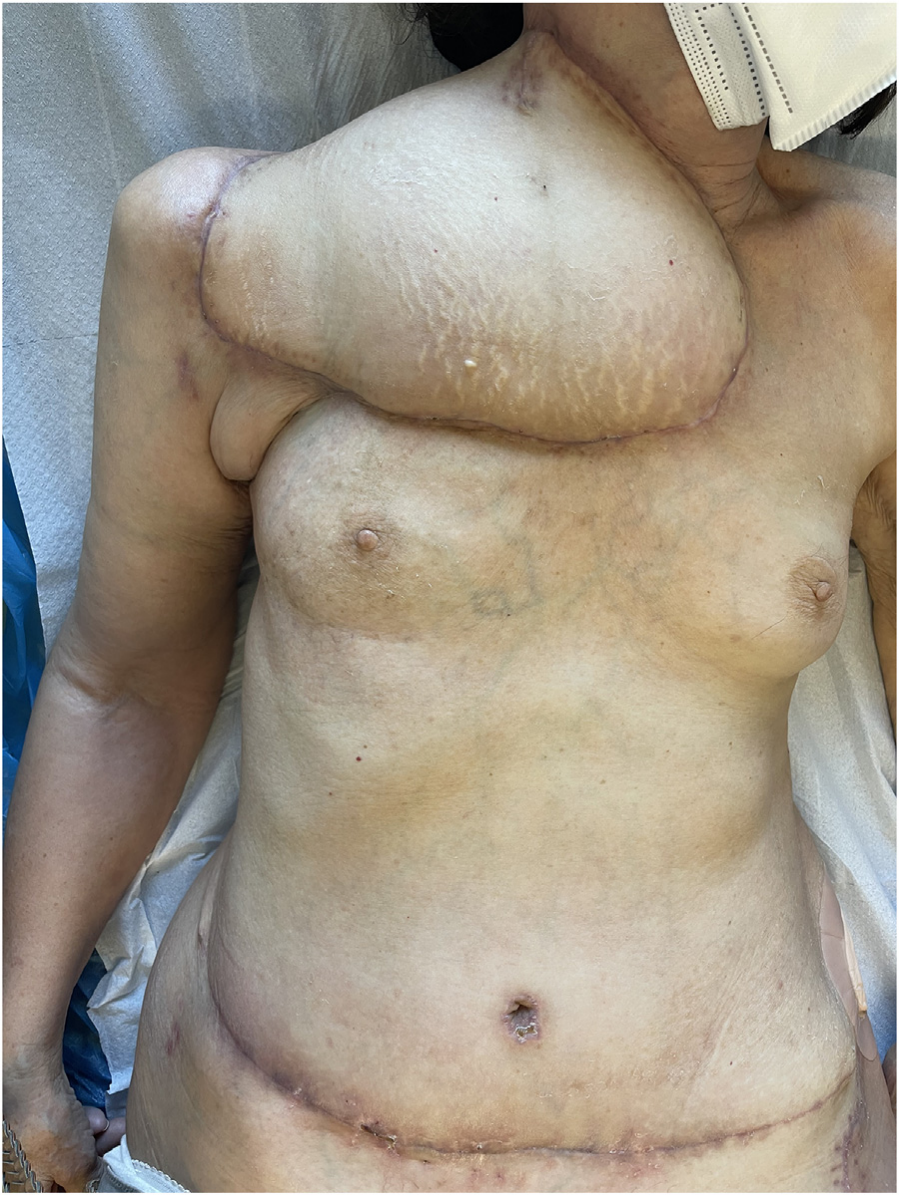
Free bipedicled DIEP flap reconstruction.

Patient Example 1: A 69-year-old female patient presented with a recurrent giant myxofibrosarcoma in the right supraclavicular and subclavicular area after three previous operations at other departments. Due to the unavailability of local flap options and significant fibrosis of neck vessels from prior radiation, a bipedicled free DIEP flap (Deep Inferior Epigastric Perforator) was used to cover the defect after wide excision of the tumor. The microanastomosis of left inferior epigastric vessels was successfully performed to the left internal mammary vessels and the right inferior epigastric vessels to the right subclavian vessels (Figure 1, Figure 2, Figure 3) . The use of free flaps for recurrent sarcomas, especially in challenging anatomical locations or previously irradiated fields, is supported by literature due to their ability to introduce healthy, well-vascularized tissue into compromised areas.

**Figure 4:**
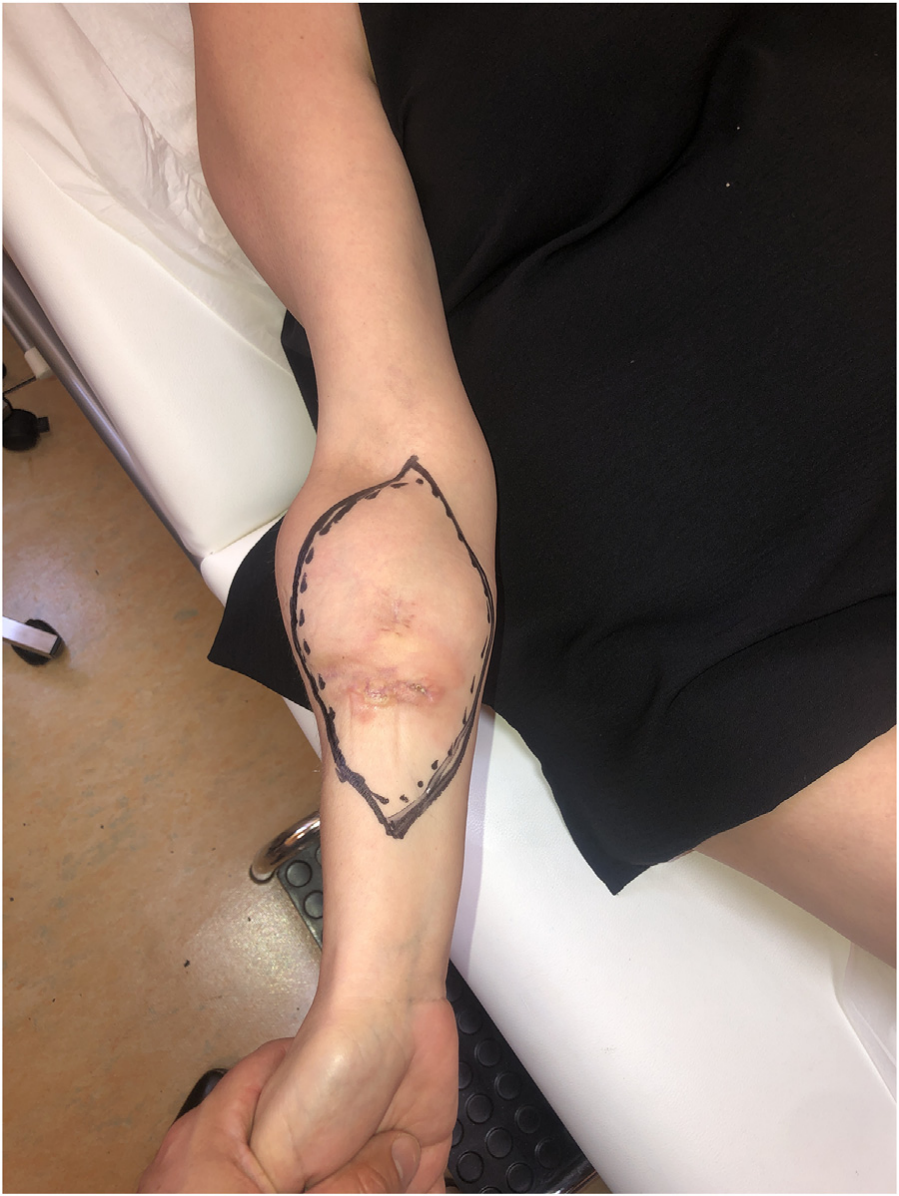
A 12×8 cm forearm synovial sarcoma.

**Figure 5:**
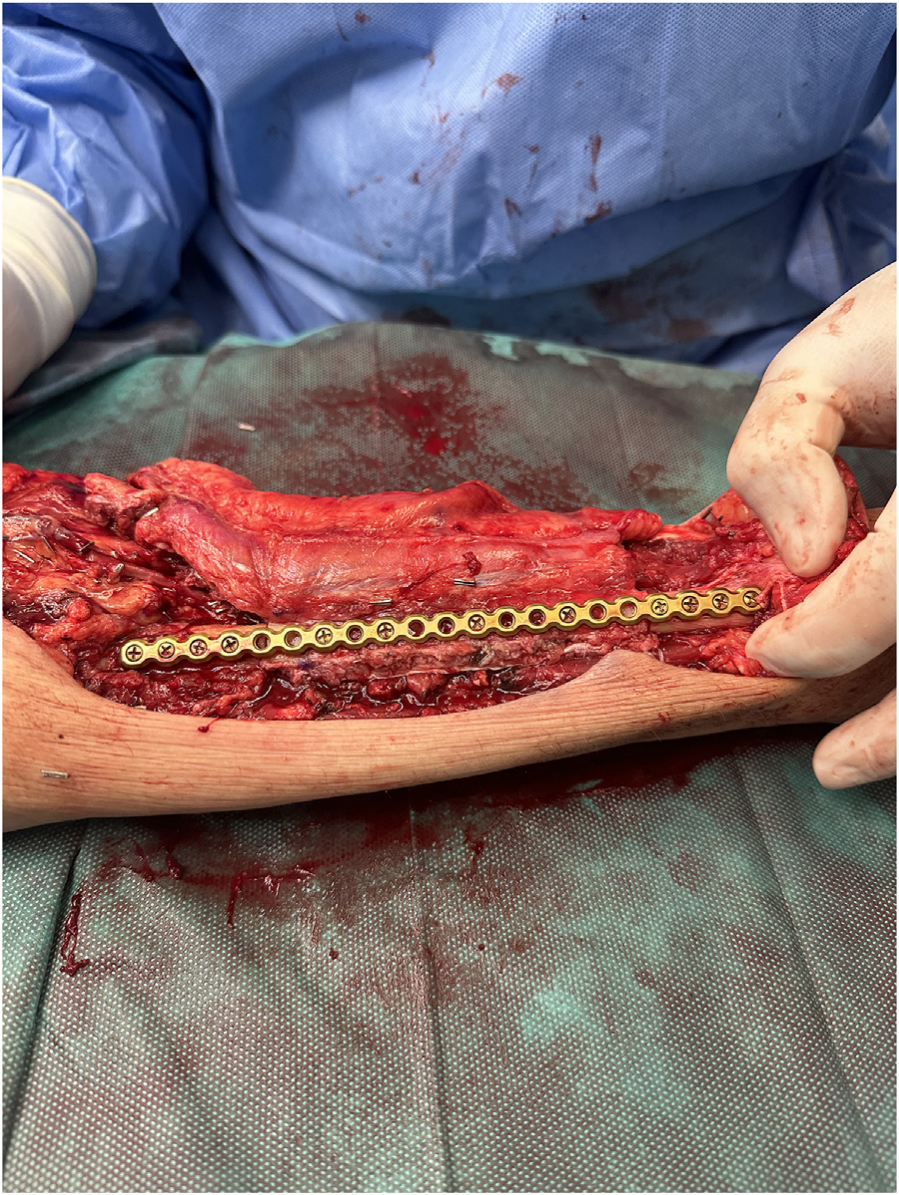
Forearm reconstruction using a vascularized osteocutaneous free fibula flap, with subsequent suturing of the extensor tendons to a fibular plate.

**Figure 6:**
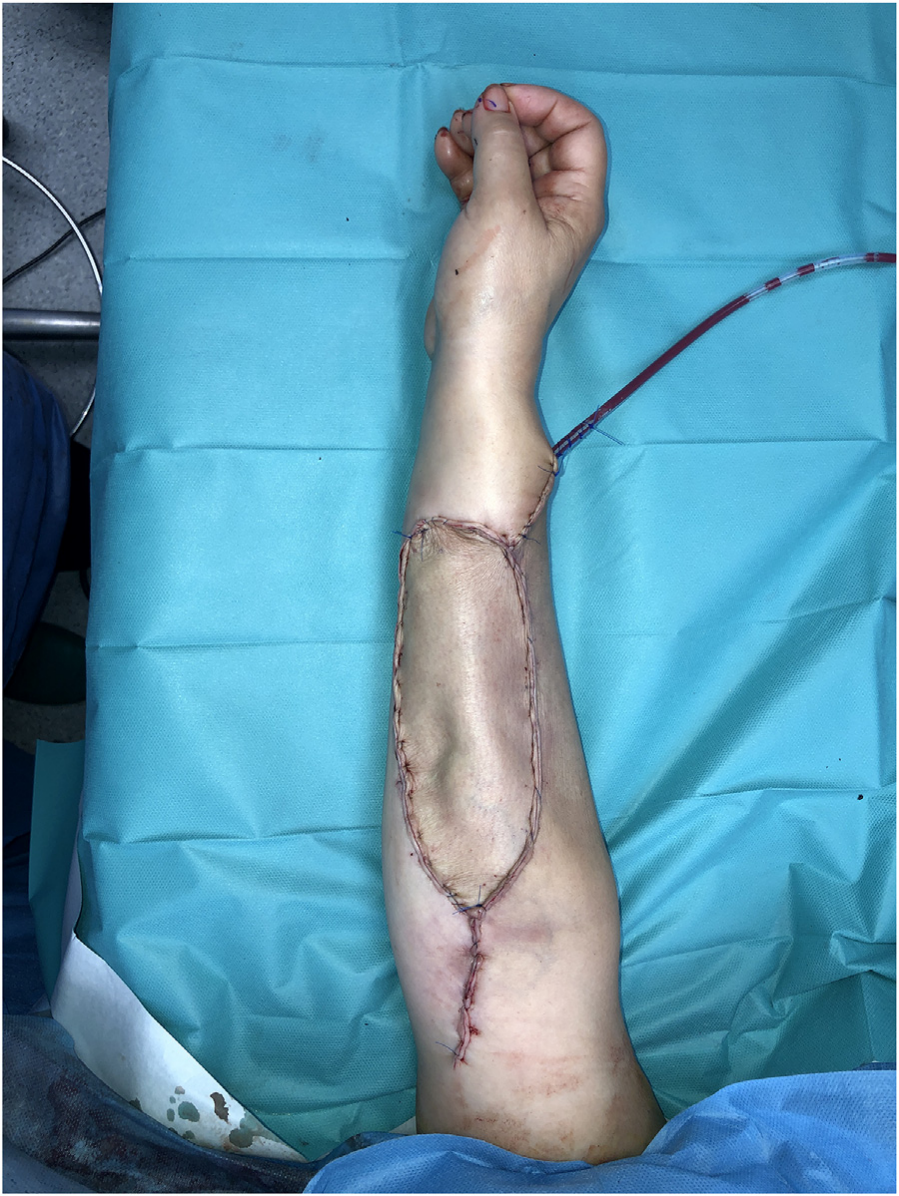
Free fibula osteocutaneous flap reconstruction.

Patient Example 2: A 35-year-old patient with a 12×8 cm forearm synovial sarcoma underwent a 20 cm radius excision, including skin and the flexor compartment. The extensive defect was reconstructed using a vascularized osteocutaneous free fibula flap, with subsequent suturing of the extensor tendons to a fibular plate (Figure 4, Figure 5, Figure 6). Free fibula flaps are well-documented for reconstructing large bone defects in the upper extremity, including those involving the forearm after sarcoma resections, especially when significant bone loss necessitates microvascular bone transfer.

**Figure 7:**
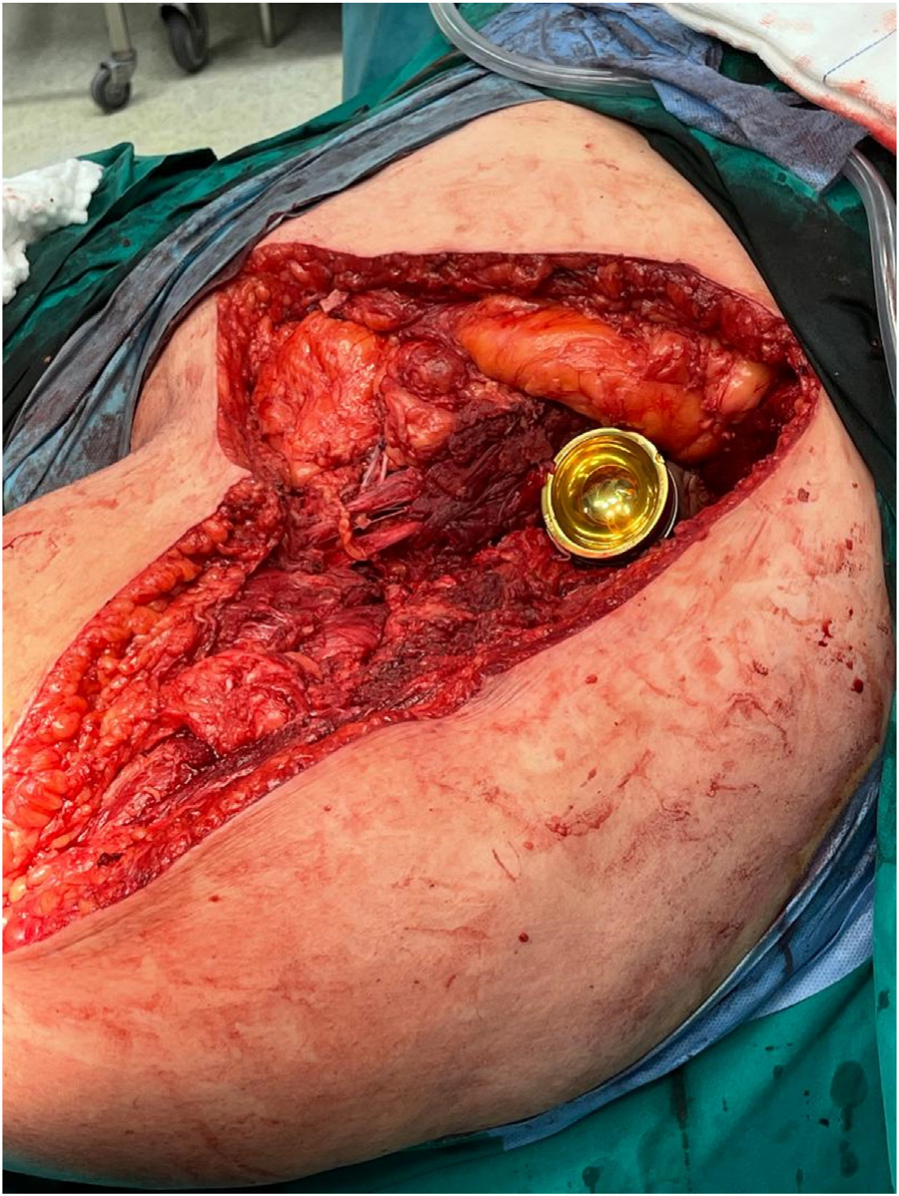
Internal hemipelvectomy for a pelvic chondrosarcoma and hip reconstruction with a “Lumic” pedestal cup.

**Figure 8:**
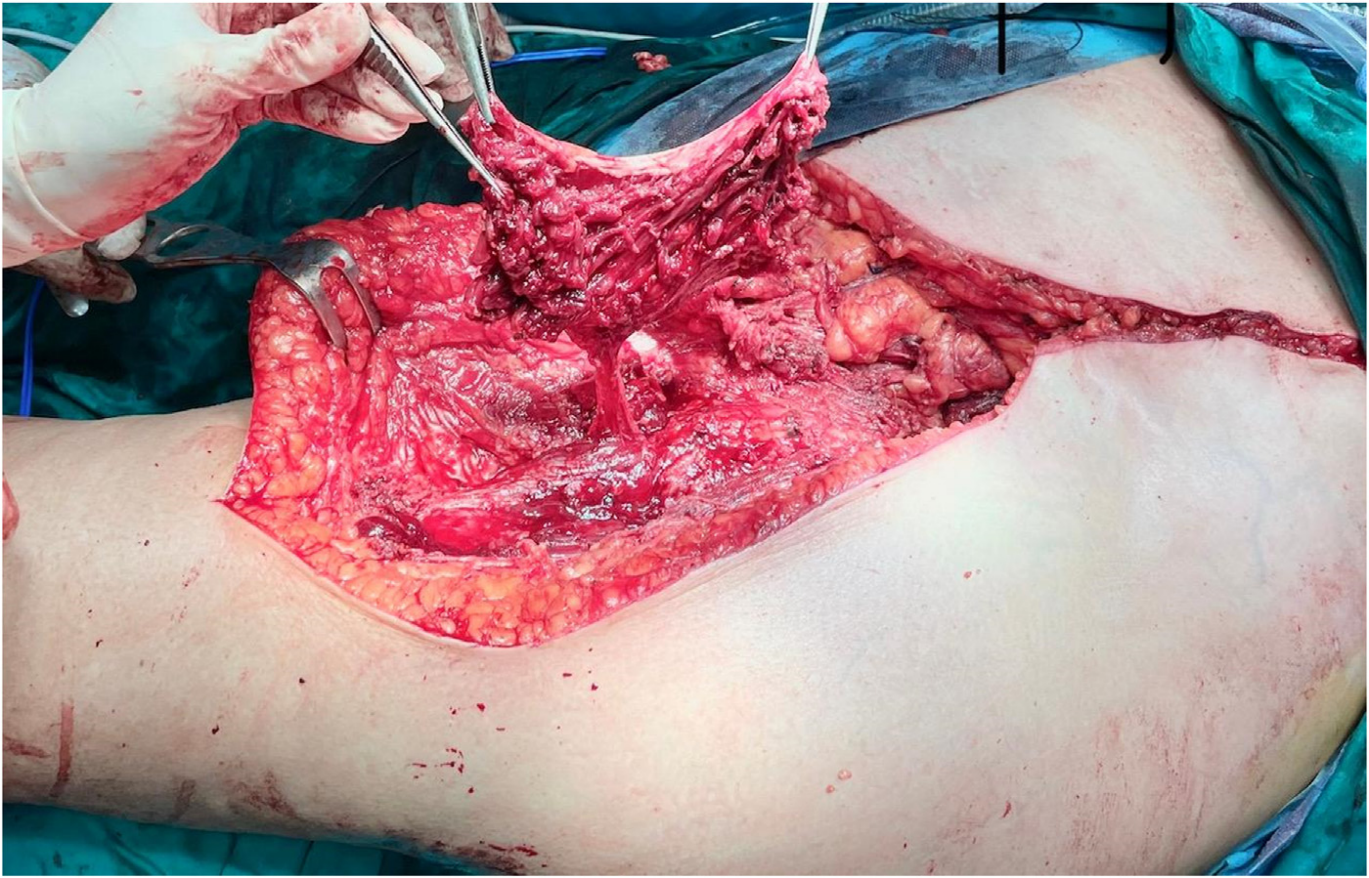
“Lumic” prosthetic reconstruction covered by pedicled vastus lateralis flap.

**Figure 9:**
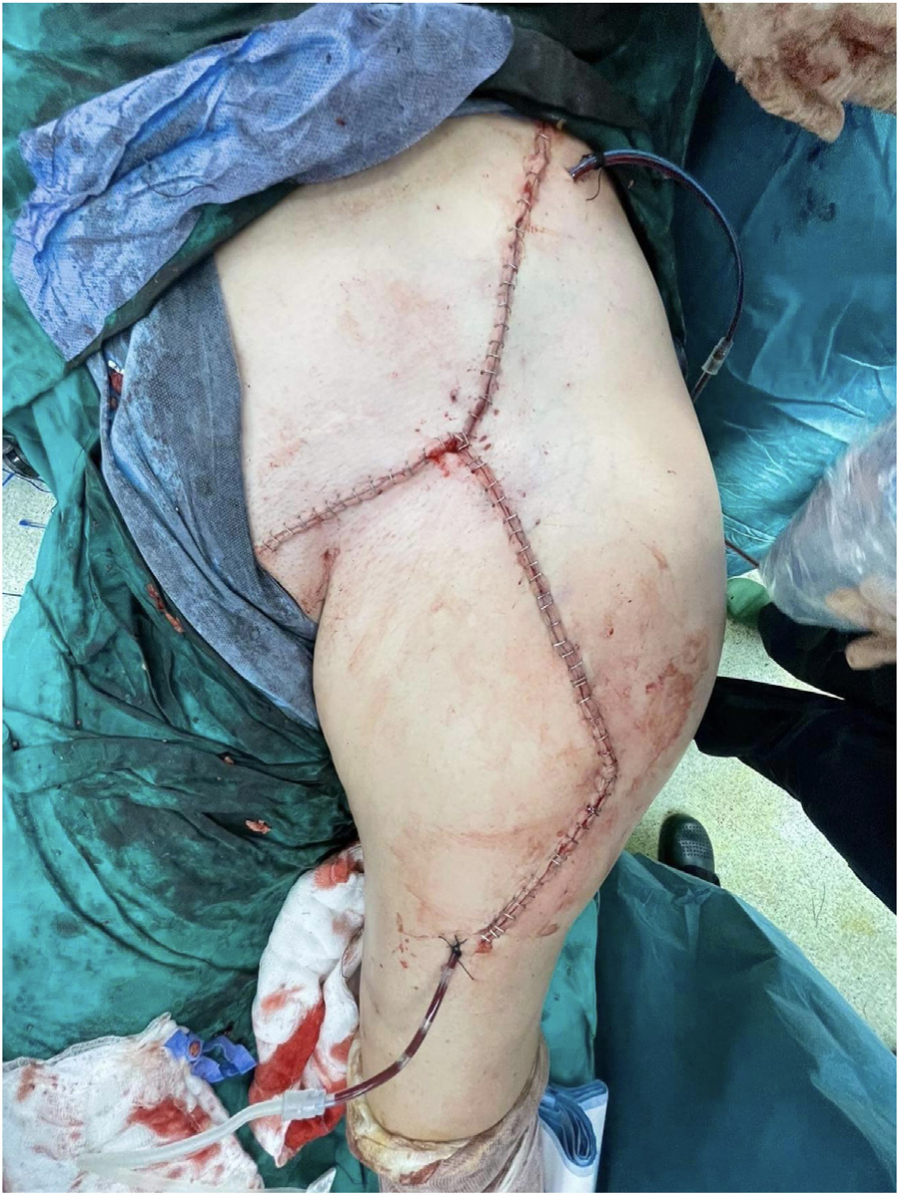
Direct closure of the defect.

Patient Example 3: A 55-year-old female patient with an iliac chondrosarcoma grade 2, measuring 11×8.6 cm, underwent hemipelvectomy type 1–3 with “lumic” reconstruction. The prosthesis was covered with a pedicled vastus lateralis flap, followed by direct skin closure (Figure 7, Figure 8, Figure 9). This illustrates the use of robust local muscle flaps for coverage following complex pelvic resections and prosthetic implantation.

**Figure 10:**
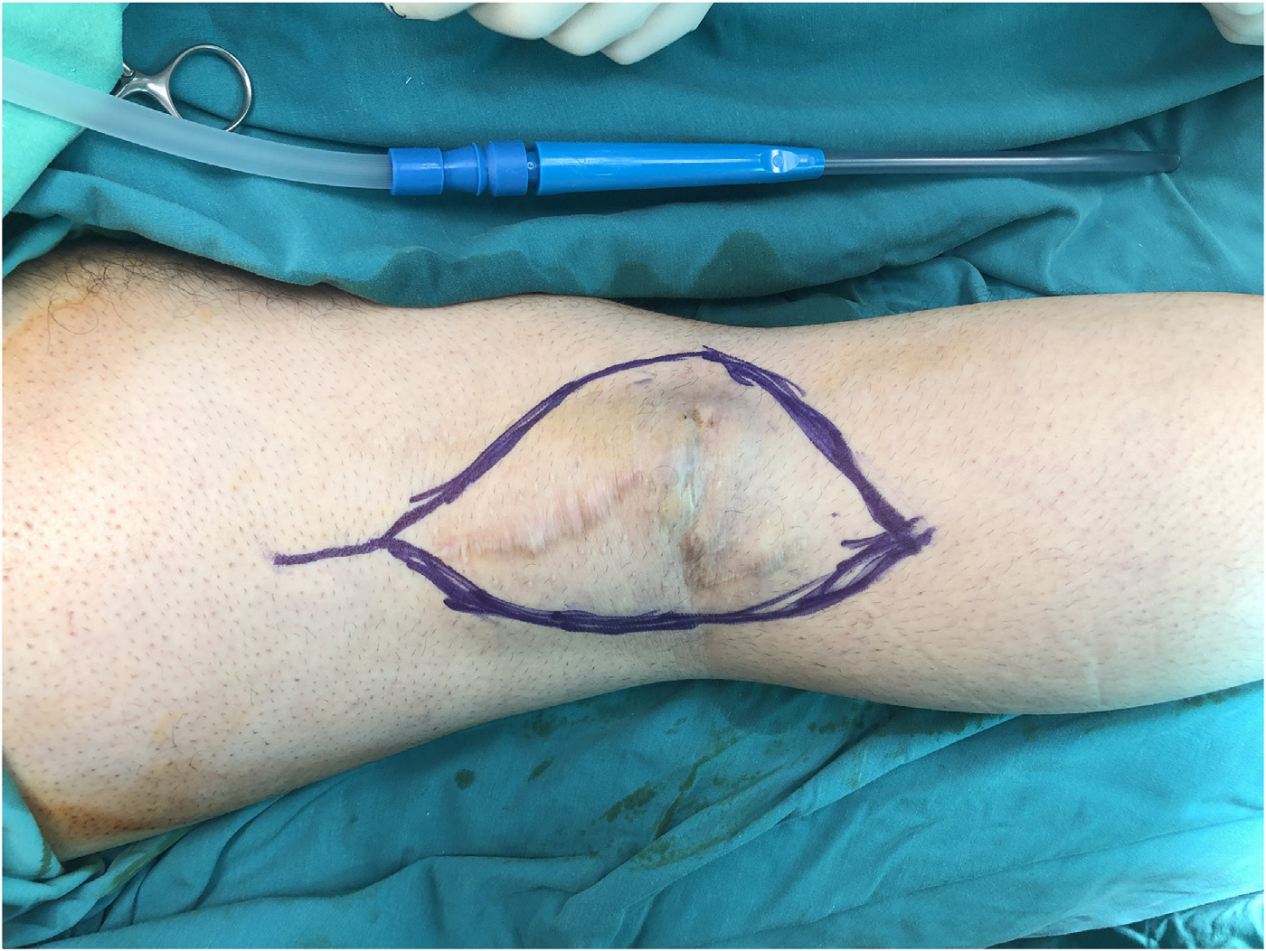
Popliteal chondrosarcoma.

**Figure 11:**
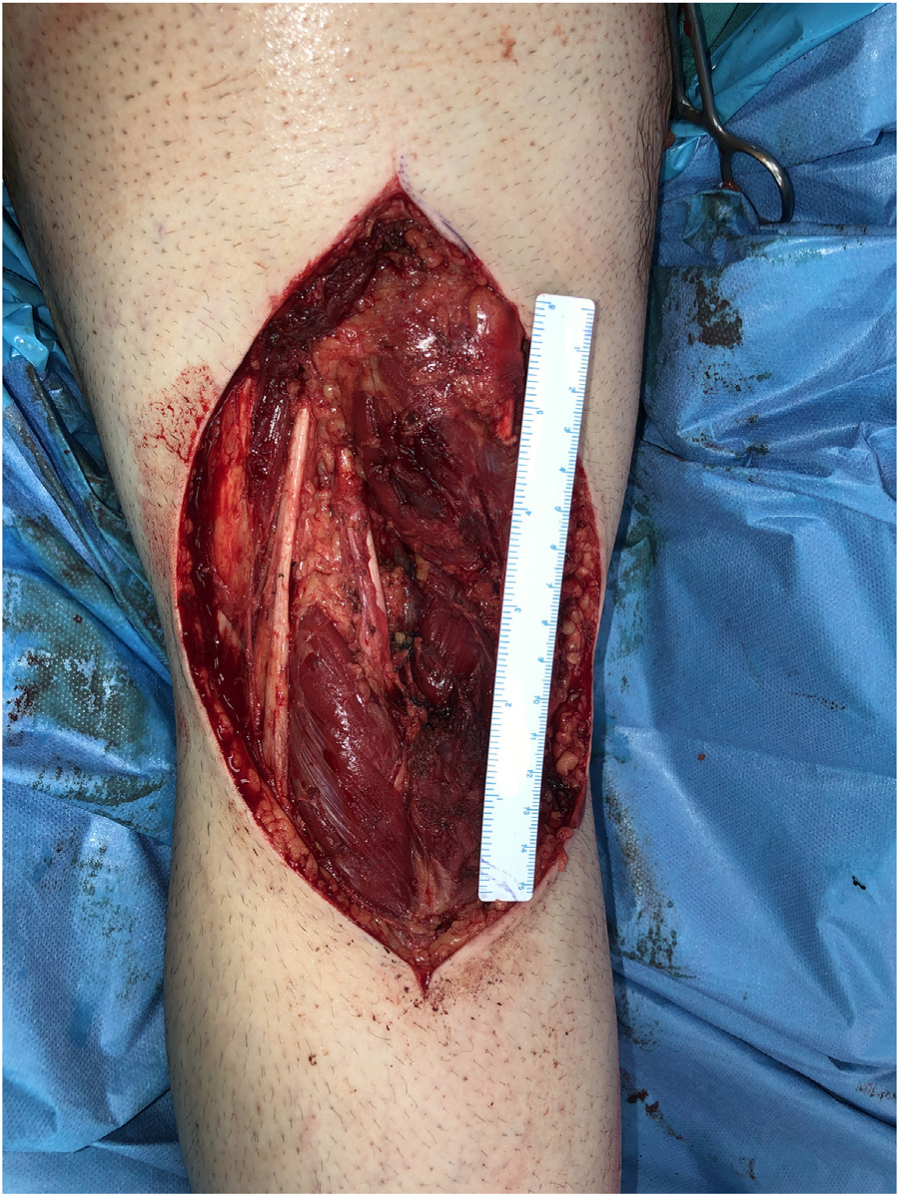
Wide surgical excision of polpliteal chondrosarcoma.

**Figure 12:**
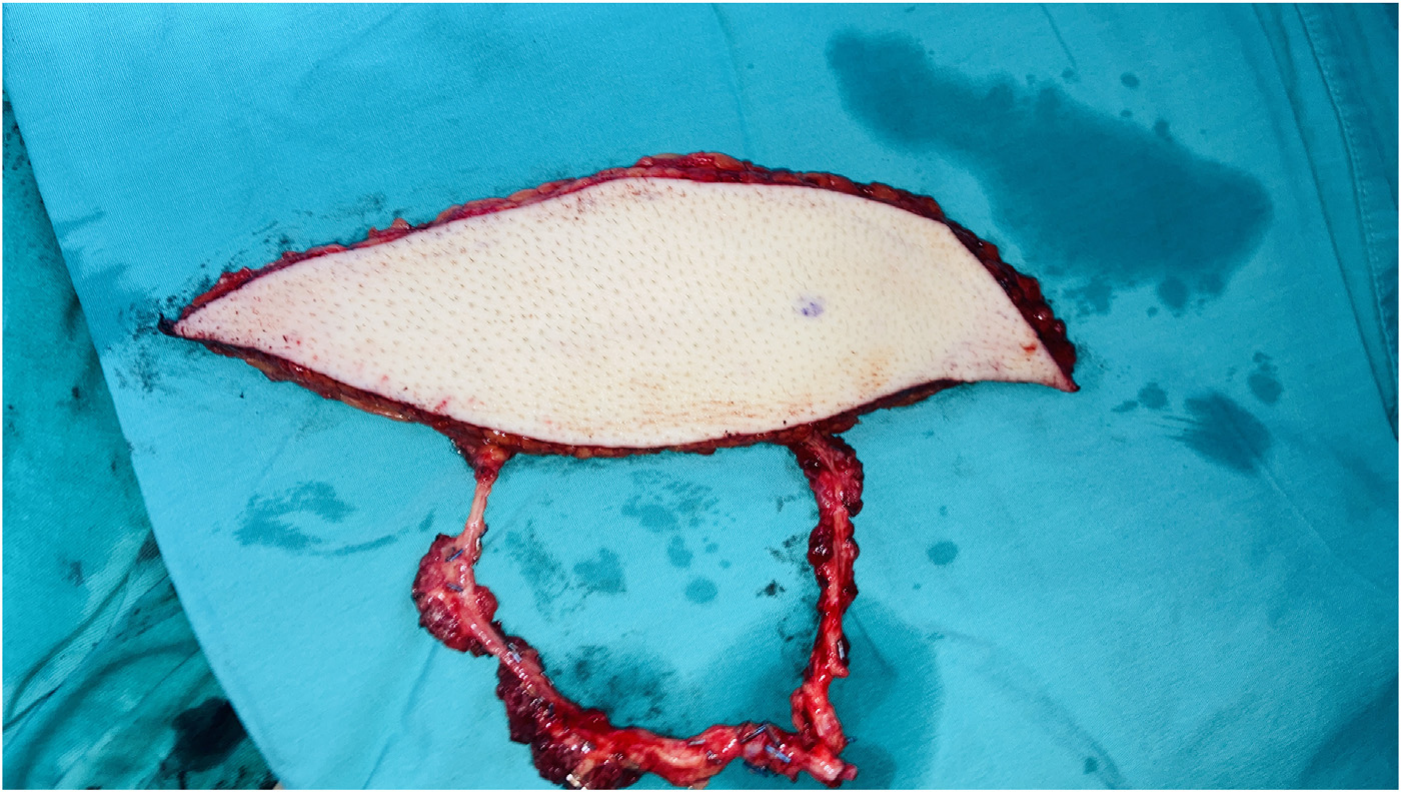
A modified type III free ALT flap.

**Figure 13:**
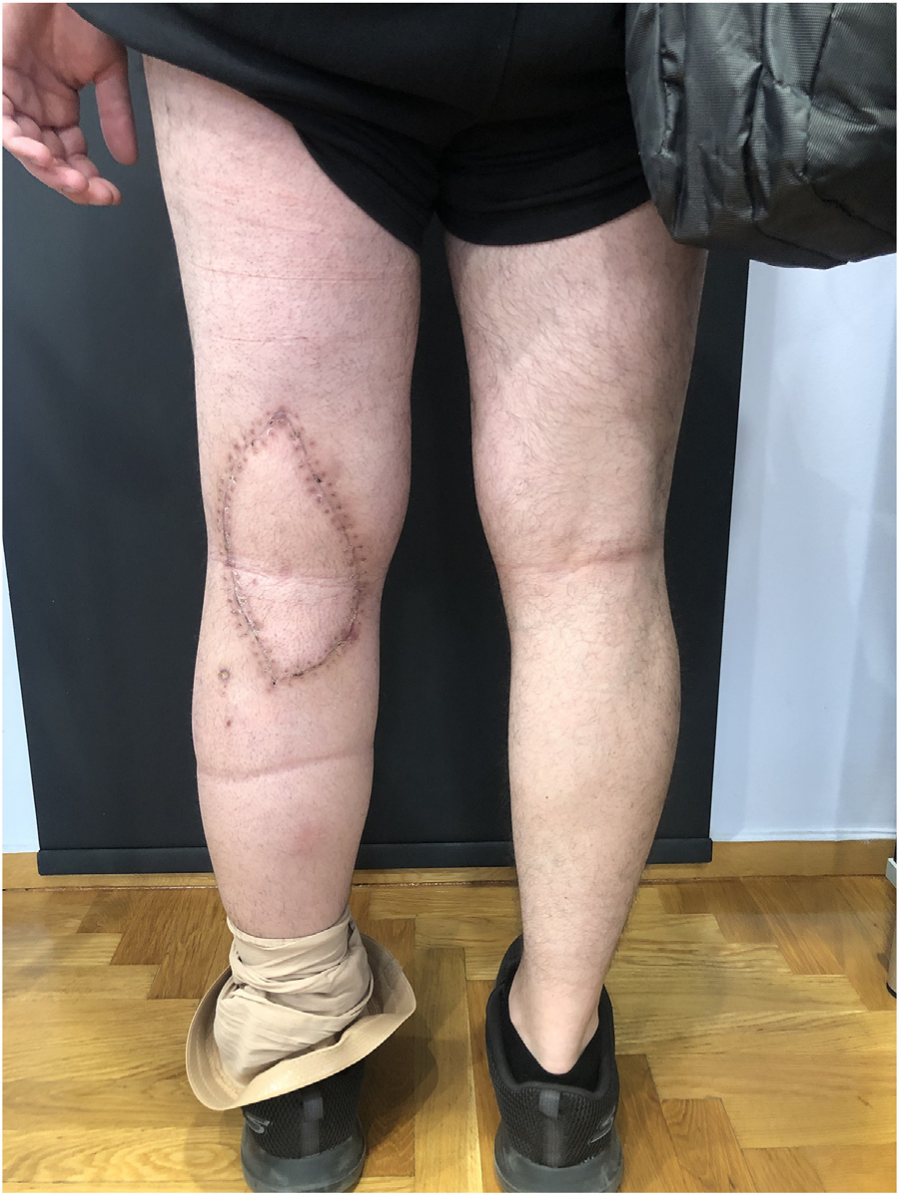
Postoperative result after wide excision of popliteal chondrosarcoma reconstructed with a free ALT flap.

Patient Example 4: Another patient 52-year-old presented with a popliteal chondrosarcoma measuring 15×11 cm. Following wide surgical excision, a type 3 free ALT flap was used for reconstruction. This was a modified type 3 flap, as the surgical anatomy revealed two perforators emerging from the medial branch of the profunda artery to the rectus femoris muscle, ultimately supplying the skin. Microanastomosis of the flap vessels was performed end-to-end to the medial sural vessels (Figure 10, Figure 11, Figure 12, Figure 13). ALT flaps are versatile for lower limb reconstruction, with variations in perforator anatomy often encountered and managed.

### Postoperative outcomes

There were no perioperative deaths. Overall flap survival was 100%, with two cases (8.7%) of partial flap necrosis managed conservatively. Minor wound complications occurred in four patients (17.4%), including marginal dehiscence (*n* = 2) and superficial infection (*n* = 2), all resolved with local care and antibiotics.

All resections in our series achieved R0 margins, with the exception of two patients with chordoma. In these two specific cases, complete R0 resection was initially not achieved due to the anatomical location and extent of the tumor. For these patients, the approach involved further surgical excision after plastic surgery reconstruction to optimize tumor clearance, followed by advancement of the previous flap and adjuvant radiotherapy to address any remaining microscopic disease.

Mean hospital stay was 10.4 days (range 6–21). No flap failures or reoperations for total flap loss were recorded.

Adjuvant therapy was administered in 15 patients (65.2%), including radiotherapy (*n* = 9) and combined chemoradiotherapy (*n* = 6). The median time to adjuvant treatment was 32 days.

At a median follow-up of 36 months (range 12–72), local recurrence occurred in three patients (13%), and distant metastasis in two (9%), both consistent with tumor histology (high-grade liposarcoma and chondrosarcoma). Limb salvage was achieved in 21 of 23 patients (91.3%).

Representative cases included:

All reconstructions achieved durable coverage and satisfactory function, with acceptable donor-site morbidity.

## Discussion

The management of musculoskeletal sarcomas requires a highly coordinated orthoplastic strategy to balance oncologic safety with optimal functional and aesthetic outcomes. Our institutional experience supports the critical role of plastic reconstructive surgery as part of an integrated, multidisciplinary framework. Nearly half of all sarcoma resections at our department required complex reconstruction, emphasizing the importance of immediate availability of reconstructive expertise in high-volume sarcoma centers. The predominance of thigh and pelvic tumors in our series reflects typical sarcoma distribution patterns.^4^^,^^5^^,^^9^ Pedicled flaps were the most commonly employed due to their reliability, robust vascularity, and reduced operative time.^23^^,^^27^ Free flaps, including ALT and DIEP flaps, were reserved for large, irradiated, or composite defects where local options were limited—consistent with international recommendations.^22^^,^^26^ Our findings reinforce previous evidence that one-stage orthoplastic reconstruction yields comparable or improved wound-healing outcomes compared with staged procedures, without increasing morbidity.^14^^,^^22^ Early definitive reconstruction enabled timely adjuvant therapy in over 90% of cases, which is critical to optimizing oncologic control.

Wound complications in this cohort (17.4%) were within the range reported in recent literature (10–25%).^14^^,^^16^^,^^20^^,^^21^ The absence of total flap failure underscores the value of multidisciplinary preoperative planning, careful flap selection, and adherence to microsurgical principles. The 91% limb-salvage rate achieved in this series is consistent with other contemporary reports, supporting the orthoplastic approach as a cornerstone of modern musculoskeletal oncology.^13^^,^^20^^,^^21^

Two patients with sacrum chordomas needed further surgical excision to optimize tumor clearance, followed by adjuvant radiotherapy. Jonathan I. Leckenby et al. proposed that staged approaches can be necessary in specific clinical scenarios.^29^

Nevertheless, this study has limitations, including its retrospective nature, moderate sample size, and single-center design. However, given the inherent rarity of musculoskeletal tumors, even at a national level, the patient sample in our retrospective case series represents a significant cohort within our specific geographical and clinical context, reflecting real-world practice within a centralized referral setting. Our center serves a leading national referral center for musculoskeletal tumors. This moderate sample size, when viewed against the background of our patient population and the rarity of the disease, provides valuable insights into the practical implementation and outcomes of the orthoplastic approach in a real-world, highly specialized setting. Future multicenter prospective studies across Europe are needed to refine reconstructive algorithms, validate outcome measures, and determine long-term functional and oncologic benefits.

## Conclusion

Plastic and reconstructive surgery is indispensable in the management of musculoskeletal tumors, particularly within a multidisciplinary orthoplastic framework. Tailored reconstructive planning based on defect characteristics, prior therapy, and anatomical location enables both radical oncologic resection (adequate surgical margins) and optimal functional preservation by providing robust reconstructive solutions. Our institutional experience demonstrates that coordinated, one-stage reconstruction facilitates early adjuvant therapy, reduces complications, and supports limb salvage in the majority of patients. Continued collaboration between orthopedic oncologists and reconstructive surgeons is essential to advance patient-centered outcomes and develop standardized, evidence-based protocols for sarcoma reconstruction.

## Funding

None.

## Ethical approval

Not required.

## Declaration of Competing interest

None declared.

## References

[1] Holt G.E., Wilson R.J., Mesko N.W., Cipriano CA. (2023). Soft-Tissue Masses: A Visual Guide to the Good, the Bad, and the Ugly. Instr Course Lect.

[2] Westhovens R., Dequeker J. (2003). Musculoskeletal manifestations of benign and malignant tumors of bone. Curr Opin Rheumatol.

[3] Bashaireh K.M., Alorjani M., Jahmani R.A. (2021 Mar). Primary Bone Tumors in North of Jordan. J Epidemiol Glob Health.

[4] Gronchi A., Miah A.B., Dei Tos A.P., ESMO Guidelines Committee, EURACAN and GENTURIS (2021 Nov). Soft tissue and visceral sarcomas: ESMO-EURACAN-GENTURIS Clinical Practice Guidelines for diagnosis, treatment and follow-up. Ann Oncol.

[5] Burningham Z., Hashibe M., Spector L., Schiffman JD. (2012 Oct 4). The epidemiology of sarcoma. Clin Sarcoma Res.

[6] Al-Hashimi M.M., Warttan H.A. (2021 Mar 12). Modelling count data with an excess of zero values applied to childhood bone tumour incidence in Iraq. Geospat Health.

[7] Solooki S., Vosoughi A.R., Masoomi V. (2011 Oct). Epidemiology of musculoskeletal tumors in Shiraz, south of Iran. Indian J Med Paediatr Oncol.

[8] Hosseini H., Heydari S., Hushmandi K., Daneshi S., Raesi R. (2025 Feb 21). Bone tumors: a systematic review of prevalence, risk determinants, and survival patterns. BMC Cancer.

[9] Alkazemi B., Ghazawi F.M., Lagacé F., Nechaev V., Zubarev A., Litvinov IV. (2023 Jun 9). Investigation of the Incidence and Geographic Distribution of Bone and Soft Tissue Sarcomas in Canada: A National Population-Based Study. Curr Oncol.

[10] Fabiano S., Contiero P., Barigelletti G. (2020). Epidemiology of Soft Tissue Sarcoma and Bone Sarcoma inItaly: Analysis of Data from 15 Population-Based Cancer Registries. Sarcoma.

[11] Sevimli R. Distribution and evaluation of primary bone and soft tissue tumors admitted from Malatya province and surrounding provinces. 10.5455/medscience.2017.06.8619

[12] Soghi A., Aarabi M., Sedaghat S.M. (2023 Feb 1). Incidence and Temporal Variations of Bone and Soft Tissue Cancers in the Golestan Province, Northern Iran, 2004-2016. Arch Iran Med.

[13] Thomas B., Bigdeli A.K., Nolte S. (2022 Sep 2). The Therapeutic Role of Plastic and Reconstructive Surgery in the Interdisciplinary Treatment of Soft-Tissue Sarcomas in Germany-Cross-Sectional Results of a Prospective Nationwide Observational Study (PROSa). Cancers (Basel).

[14] Angelini A., Tiengo C., Sonda R., Berizzi A., Bassetto F., Ruggieri P. (2020 Dec 14). One-Stage Soft Tissue Reconstruction Following Sarcoma Excision: A Personalized Multidisciplinary Approach Called "Orthoplasty". J Pers Med.

[15] Koesters E.C., Chang DW. (2023 Aug 30). Radiation and free flaps: what is the optimal timing?. Gland Surg.

[16] Honig R.L., Tibbo M.E., Mallett K.E. (2020). Outcome of Soft-tissue Reconstruction in the Setting of Combined Preoperative and Intraoperative Radiotherapy for Extremity Soft-tissue Sarcomas. Anticancer Research.

[17] Thariat J. et al. Reconstructive flap surgery in head and neck cancer patients: an interdisciplinary view of the challenges encountered by radiation oncologists in postoperative radiotherapy. Front. Oncol. 14:1379861. 10.3389/fonc.2024.1379861 PMC11043495 38665951

[18] Walker E.A., Song A., Murphey M.D. (2010). Magnetic Resonance Imaging of Soft-Tissue Masses. Radiologic Clinics of North America.

[19] Sedaghat S., Sedaghat M., Meschede J. (2021). Diagnostic value of MRI for detecting recurrent soft-tissue sarcoma in a long-term analysis at a multidisciplinary sarcoma center. BMC Cancer.

[20] Strönisch A., Märdian S., Flörcken A. (2023). Centralized and Interdisciplinary Therapy Management in the Treatment of Sarcomas. Cancers.

[21] Blay J.-Y., Soibinet P., Penel N. (2017). Improved survival using specialized multidisciplinary board in sarcoma patients. Annals of Oncology.

[22] Moreira-Gonzalez A., Djohan R., Lohman R. (2010 Mar). Considerations surrounding reconstruction after resection of musculoskeletal sarcomas. Cleve Clin J Med.

[23] Radtke C., Panzica M., Dastagir K., Krettek C., Vogt PM. (2016 Jan 13). Soft Tissue Coverage of the Lower Limb following Oncological Surgery. Front Oncol.

[24] Götzl R., Sterzinger S., Arkudas A. (2020 Nov 26). The Role of Plastic Reconstructive Surgery in Surgical Therapy of Soft Tissue Sarcomas. Cancers (Basel).

[25] Quildrian S.D., Nardi W.S., Vega M.G., Chapela J.A. (2024). The role of free flap reconstruction after resection of extremity and trunk soft tissue sarcomas. Results of two referral centers in Argentina. Clinical Surgical Oncology.

[26] Krauss S., Goertz O., Pakosch-Nowak D. (2021 May). Microvascular tissue transfer after the resection of soft tissue sarcomas. J Plast Reconstr Aesthet Surg.

[27] Elswick S.M., Wu P., Arkhavan A.A. (2019 Aug). A reconstructive algorithm after thigh soft tissue sarcoma resection including predictors of free flap reconstruction. J Plast Reconstr Aesthet Surg.

[28] Parikh R.P., Sacks JM. (2021 Apr). Lower Extremity Reconstruction After Soft Tissue Sarcoma Resection. Clin Plast Surg.

[29] Leckenby J.I., Deegan R., Grobbelaar AO. (2018 Jan). Complex Reconstruction After Sarcoma Resection and the Role of the Plastic Surgeon: A Case Series of 298 Patients Treated at a Single Center. Ann Plast Surg.

